# The impact of the new model of day care on the mental health status and cognitive functions of patients with disabilities treated in day medical care homes in Poland, 2017-2023

**DOI:** 10.3389/fpsyt.2024.1403028

**Published:** 2024-07-23

**Authors:** Leszek Warsz, Mateusz Jankowski, Agata Andrzejczyk, Mariusz Gujski

**Affiliations:** ^1^ Department of Public Health, Medical University of Warsaw, Warsaw, Poland; ^2^ Medical and Diagnostic Center, Siedlce, Poland; ^3^ Department of Population Health, Centre of Postgraduate Medical Education, Warsaw, Poland; ^4^ Faculty of Health Sciences, Medical University of Warsaw, Warsaw, Poland

**Keywords:** mental health, older adults, aging, day care, organization of health

## Abstract

**Introduction:**

Day Medical Care Home is a care and treatment institution providing day care services for people with disabilities (mainly older adults), implemented in Poland since 2015. This study aimed to analyze the impact of the new model of day care on the mental health status and cognitive functions of older adults with disabilities treated in Day Medical Care Homes in Poland, 2017-2023.

**Methods:**

Admission and discharge reports of 949 patients treated in Day Medical Care Homes between 2017 and 2023 were analyzed. Geriatric Depression Scale (GDS-15) and Hamilton Rating Scale for Depression (HAM-D) were used to assess mental health. Cognitive functions were assessed using Mini–Mental State Examination (MMSE).

**Results:**

The mean age was 74.3 ± 8.5 years, 76.6% were women. and 62.6% lived in rural areas. The percentage of patients with moderate or severe depression measured with a GDS-15 score decreased from 58.3% on admission to 34.6% on discharge (p<0.001). The percentage of patients with depressive disorders measured with HAM-D score decreased from 48.3% on admission to 37.2% on discharge (p<0.001). The percentage of patients with dementia or cognitive disorders measured with the MMSE score decreased from 54.3% on admission to 40.5% on discharge (p<0.001). Improvement in mental health status and cognitive functions was observed in all sociodemographic groups.

**Conclusion:**

Day Medical Care Home is an effective model of day care that improves the mental health status and cognitive functions of older adults with disabilities.

## 1 Introduction

Falls, cognitive disorders, depression, and delirium are major geriatric syndromes that have a significant impact on the health status of older adults aged 60 years and over (1, 2) The global prevalence of major depression in older adults is estimated at 13.3%, including 11.9% among women and 9.7% among men (3). It is estimated that the prevalence of dementia increases from 1.8% among people aged 60-69 years, up to 15.1% among people aged 80-89 years, and over 35% of people aged 90 and over (4). Mental health disorders often coexist with cognitive disorders (2).

Cognitive disorders have a negative impact on the health of people affected by them, as well as evoke measurable medical and social costs (5). The presence of depressive symptoms may worsen the course of comorbidities (e.g., diabetes and cardiovascular diseases) and negatively affect patients’ medication adherence (6, 7). Living alone, which is common among older adults, has been associated with increased mortality risk (8).

Healthy aging is defined as maintaining a functional ability that enables older patients to meet their needs and contribute to society within their environment (9). Reducing the risk of non-communicable chronic diseases, improving physical activity and mental capacity as well as delaying care dependency are key objectives of public health interventions on healthy aging (10).

Population aging, especially in high-income countries prompted public authorities to develop strategies for healthy aging and institutions that may provide care for older people (11, 12). Public policies on healthy aging should include interventions for physical health as well as mental well-being (5, 11). Providing care services for older people is an interdisciplinary challenge for healthcare professionals as well as social care experts.

Poland is a high-income European country with a rapidly aging population (13). Between 2022 and 2050, the proportion of people aged 60 and over in Poland will increase from 26% to 40% (13). In Poland, senior care was traditionally provided in the informal form in the family setting (14). However, population aging prompted public authorities in Poland to develop a new form of senior care, that will support healthy aging and reduce the risk of hospitalization of older patients.

In 2015, public authorities in Poland introduced a new form of day care, so-called Day Medical Care Homes (15, 16). Day Medical Care Home is a day care and treatment institution providing day care services for people with disabilities (mainly older adults), especially those at risk of hospitalization or rehospitalization (15, 16). Daily Medical Care Homes were co-funded by the European Social Fund under the Knowledge Education Development Operational Program (PO WER), implemented in cooperation with local governments (15, 16). Day Medical Care Homes offered medical care combined with activities aimed at improving the functional, mental, and cognitive status of older patients with disabilities (15). The patient’s stay at the Day Medical Care Home should range from 30 to 120 days, and during the day the patient should spend at least 4 hours under the care of the facility (15, 16). The Day Medical Care Home offered services provided by doctors (including specialists in geriatrics and medical rehabilitation), nurses, medical caregivers, occupational therapists, and psychologists or psychotherapists (15, 16). The scope of services offered by Day Medical Care Homes included: patient education in the field of self-care, advice on the selection of appropriate medical devices, motor improvement and rehabilitation, stimulation of cognitive processes, occupational therapy, and education of the patient’s family and caregivers on senior care (15, 16). The scope of services was individually selected according to the patient’s health condition and assessed upon admission to Day Medical Care Homes using a set of scales and questionnaires. Each patient participated in occupational therapy (mainly group classes) for at least 10 hours a week (15, 16). In addition, Day Medical Care Homes offered the help of a psychologist and psychotherapist, depending on the individual needs (15, 16). During their stay at the Day Care Medical Home, patients were treated following the existing pharmacotherapy regimens recommended by specialist physicians (based on medical documentation on admission), and the only pharmacological interventions concerned drug reviews and identification of polypharmacy (15, 16). At Day Care Medical Home there was no access to a psychiatrist and no changes were made to mental health issues pharmacotherapy (15, 16). The therapeutic team evaluates the results of actions taken for each patient and makes any changes in the care plan (15).

Day Medical Care Homes were based on the day care approach, widely implemented in psychiatry (17). Senior care services offered in Day Medical Care Homes for at least 4 hours per day were designed as a cost-effective form of senior care, that should improve both the functional and mental health status of the patients covered by the care (16). However, currently, there is no scientific research on the impact of this new day care model on the mental health and cognitive functions of patients receiving this new form of care.

This study aimed to analyze the impact of the new model of day care on the mental health status and cognitive functions of older adults with disabilities treated in Day Medical Care Homes in Poland, 2017-2023.

## 2 Materials and methods

### 2.1 Study design and population

Data on patients admitted to Day Medical Care Homes (DDOM) managed by the Medical and Diagnostic Centre (CMD), Poland between 2017 and 2023 were driven from health records. The health status of patients (on-admission and on-discharge) was assessed with 11 different scales (in line with the requirements of the funding institution – European Union (EU) funding program), including 3 scales on mental health: Geriatric Depression Scale (GDS-15) (18), Hamilton Rating Scale for Depression (21-items) (19), and Mini–Mental State Examination (MMSE) (20). Trained psychologists carried out patients’ assessments with the GDS-15 scale, HAM-D scale, and MMSE on admission and discharge. Health status of the patients was assessed with the New York Heart Association (NYHA) scale by a doctor. All procedures were carried out following guidelines expressed in the Declaration of Helsinki and the protocol was approved by the Ethics Committee at the Medical University of Warsaw, Poland (approval number: AKBE/298/2023; 13 Nov 2023).

The following medical requirements were in place for admission to Day Medical Care Homes:

Inclusion criteria:

• patients with a Barthel Index score between 40 and 65 points (21),

• patients immediately after hospitalization who require care to reduce the risk of independence as well as medical support and health education;

• patients who were hospitalized within 12 months and people who are at high risk of hospitalization in the near future and whose health condition - verified based on the Barthel scale - indicates dependence (Barthel Index score between 40 and 65 points).

Exclusion criteria:

• individuals using health care services and care as part of long-term care, palliative and hospice care, and therapeutic rehabilitation, in the conditions of a center or day ward, and stationary conditions;

• individuals for whom the primary indication for care would be advanced mental illness or addiction (using health services in the field of psychiatric care and addiction treatment).

### 2.2 Measures

Sociodemographic characteristics of patients admitted to Day Medical Care Homes included sex, age, and place of residence. Data on the duration of care were calculated based on admission and discharge dates. The mental health status of patients was assessed twice: on admission and discharge. Geriatric Depression Scale (GDS-15) (18, 22) and Hamilton Rating Scale for Depression (21 items) (19) were used to assess the occurrence and severity of depressive symptoms.

The Geriatric Depression Scale (GDS-15) short form was mandatory for patients aged 65 and over and voluntary for those under 65 years (18, 22). The maximum score was 15 points, with the following cut-off points: 0-5 no depressive disorder (normal score); 6-10 moderate depression; 11-15 severe depression (18, 22).

Hamilton Rating Scale for Depression (HAM-D), full version with 21 items was mandatory for patients aged under 65 and voluntary for patients aged over 65 years (19). The following cut-off points were used following the guidelines of the American Psychological Association: 0-7 points – no depressive disorders; 8-13 points – mild depression; 14-18 points – moderate depression; 19-22 points – severe depression; 23 or more points – very severe depression (19).

Mini-Mental State Examination (MMSE) was used to assess patients’ cognitive status and the occurrence of dementia (23). This scale was mandatory for all patients regardless of age. The maximum score was 30 points, with the following cut-off points: 0-10 profound dementia; 11-18 medium-grade dementia, 19-23 mild dementia; 24-26 cognitive disorders without dementia; 27-30 correct result (no dementia or cognitive disorders) (23).

Numerical scores in GDS, Hamilton Scale, and MMSE scales were assigned to categories in line with the abovementioned cut-off points.

### 2.3 Statistical analysis

Statistical testing was completed using IBM SPSS Statistics v. 29 (USA: IBM, Armonk, NY). The distribution of categorical variables was presented with frequencies and proportions. Cross-tables along with a chi-squared test were used to compare categorical variables. A Spearman rank correlation test was used to assess the relationship between duration of care and the absolute differences on discharge and on admission in GDS-15, HAM-D, and MSSE scores. The statistical significance level was based on the criterion p < 0.05.

## 3 Results

Completed medical records on mental health status on admission and discharge were available for 949 patients with a mean age of 74.3 ± 8.5 years, 96.6% of patients were aged 60 and over, and 76.6% were women. Almost two-thirds of patients (62.6%) lived in rural areas. Over half of the patients were covered by care in Day Medical Care Homes for 41 to 50 days (51.5%). Over one-third of patients were assigned to NYHA class I on admission (36.2%), 48.3% were assigned to class II, 15.3% to class III and 2 patients were assigned on admission to NYHA class IV. Details are presented in **Table 1**.

**Table 1:** Characteristics of patients admitted to Day Medical Care Homes managed by CDM, between 2017 and 2023 (n=949).

Variable	N	%
Sex		
Women	727	76.6
Men	222	23.4
Age		
24-59	32	3.4
60-69	221	23.3
70-79	428	45.1
80-89	248	26.1
90+	20	2.1
Duration of care		
up to 40 days	135	14.2
41-50 days	489	51.5
51-80 days	94	9.9
81 days and over	231	24.3
Place of residence		
urban area	355	37.4
rural area	594	62.6
NYHA class on admission		
I	344	36.2
II	458	48.3
III	145	15.3
IV	2	0.2

### 3.1 Changes in the prevalence of depressive disorders among patients covered by care in day medical care homes

Among patients covered by care in Day Medical Care Homes, the percentage of patients with moderate or severe depression measured with a GDS-15 score decreased from 58.3% on admission to 34.6% on discharge (p<0.001) (**Table 2**). Improvement in mental health status defined as lack of depression measured by the Geriatric Depression Scale (p<0.05) was observed in all sociodemographic groups. Moreover, improvement (p<0.001) in mental health status was observed in all patients regardless of duration of care in a Day Medical Care Home (**Table 2**).

**Table 2:** Changes in the GDS score of patients covered by care in Day Medical Care Homes (n=949).

Variable	GDS-15 score on admission			GDS-15 score on discharge			
	no depression (0-5)	moderate depression (6-10)	severe depression (11-15)	no depression (0-5)	moderate depression (6-10)	severe depression (11-15)	p
	n (%)	n (%)	n (%)	n (%)	n (%)	n (%)	
**Overall**	396 (41.7)	422 (44.5)	131 (13.8)	620 (65.3)	265 (27.9)	64 (6.7)	**<0.001**
**Sex**							
women	299 (41.1)	323 (44.4)	105 (14.4)	477 (65.6)	201 (27.6)	49 (6.7)	**<0.001**
Men	97 (43.7)	99 (44.6)	26 (11.7)	143 (64.4)	64 (28.8)	15 (6.8)	**<0.001**
**Age**							
24-59	12 (37.5)	14 (43.8)	6 (18.8)	17 (53.1)	14 (43.8)	1 (3.1)	**0.001**
60-69	94 (42.5)	92 (41.6)	35 (15.8)	151 (68.3)	51 (23.1)	19 (8.6)	**<0.001**
70-79	182 (42.5)	192 (44.9)	54 (12.6)	292 (68.2)	115 (26.9)	21 (4.9)	**<0.001**
80-89	99 (39.9)	115 (46.4)	34 (13.7)	144 (58.1)	81 (32.7)	23 (9.3)	**<0.001**
90+	9 (45.0)	9 (45.0)	2 (10.0)	16 (80.0)	4 (20.0)	0 (0.0)	**0.006**
**Duration of care**							
up to 40 days	54 (40.0)	41 (30.4)	40 (29.6)	82 (60.7)	36 (26.7)	17 (12.6)	**<0.001**
41-50 days	208 (42.5)	213 (43.6)	68 (13.9)	305 (62.4)	147 (30.1)	37 (7.6)	**<0.001**
51-80 days	32 (34.0)	59 (62.8)	3 (3.2)	81 (86.2)	11 (11.7)	2 (2.1)	**<0.001**
81 days and over	102 (44.2)	109 (47.2)	20 (8.7)	152 (65.8)	71 (30.7)	8 (3.5)	**<0.001**
**Place of residence**							
urban area	167 (47.0)	123 (34.6)	65 (18.3)	226 (63.7)	99 (27.9)	30 (8.5)	**<0.001**
rural area	229 (38.6)	299 (50.3)	66 (11.1)	394 (66.3)	166 (27.9)	34 (5.7)	**<0.001**

Statistically significant values are bolded.

Among patients covered by care in Day Medical Care Homes, the percentage of patients with depressive disorders measured with HAM-D score decreased from 48.3% on admission to 37.2% on discharge (p<0.001) (**Table 3**). Improvement in mental health status defined as lack of depression measured by the Hamilton Rating Scale for Depression (p<0.05) was observed in all sociodemographic groups and regardless of duration of care in the Day Medical Care Home (**Table 3**). There was a statistically significant correlation between duration of care and absolute differences in HAM-D scores on discharge and admission (rho=-0.1; p=0.003), but there was no significant correlation between duration of stay and absolute changes in GDS-15 score (rho=0.05; p=0.2).

**Table 3:** Changes in the HAM-D score of patients covered by care in Day Medical Care Homes (n=795).

Variable	HAM-D score on admission					HAM-D score on discharge					
	no depression (0-7	mild depression (8-13)	moderate depression (14-18)	severe depression (19-22)	very severe depression(≥23)	no depression (0-7	mild depression (8-13)	moderate depression (14-18)	severe depression (19-22)	very severe depression(≥23)	p
	n (%)	n (%)	n (%)	n (%)	n (%)	n (%)	n (%)	n (%)	n (%)	n (%)	
**Overall**	411 (51.7)	325 (40.9)	48 (6.0)	7 (0.9)	4 (0.5)	499 (62.8)	284 (35.7)	11 (1.4)	0 (0.0)	1 (0.1)	**<0.001**
Sex											
women	323 (51.8)	253 (40.5)	39 (6.3)	5 (0.8)	4 (0.6)	386 (61.9)	227 (36.4)	10 (1.6)	0 (0.0)	1 (0.2)	**<0.001**
men	88 (51.5)	72 (42.1)	9 (5.3)	2 (1.2)	0 (0.0)	113 (66.1)	57 (33.3)	1 (0.6)	(0.0)	0 (0.0)	**<0.001**
Age											
24-59	14 (45.2)	15 (48.4)	2 (6.5)	0 (0.0)	0 (0.0)	17 (54.8)	14 (45.2)	0 (0.0)	0 (0.0)	0 (0.0)	**<0.001**
60-69	103 (53.9)	73 (38.2)	12 (6.3)	2 (1.0)	1 (0.5)	125 (65.4)	64 (33.5)	2 (1.0)	0 (0.0)	0 (0.0)	**<0.001**
70-79	198 (54.5)	140 (38.6)	21 (5.8)	2 (0.6)	2 (0.6)	234 (64.5)	124 (34.2)	4 (1.1)	0 (0.0)	1 (0.3)	**<0.001**
80-89	91 (46.4)	91 (46.4)	11 (5.6)	2 (1.0)	1 (0.5)	116 (59.2)	75 (38.3)	5 (2.6)	0 (0.0)	0 (0.0)	**<0.001**
90+	5 (35.7)	6 (42.9)	2 (14.3)	1 (7.1)	0 (0.0)	7 (50.0)	7 (50.0)	0 (0.0)	0 (0.0)	0 (0.0)	**0.03**
Duration of care											
up to 40 days	79 (63.2)	41 (32.8)	5 (4.0)	0 (0.0)	0 (0.0)	88 (70.4)	37 (29.6)	0 (0.0)	0 (0.0)	0 (0.0)	**<0.001**
41-50 days	240 (59.6)	139 (34.5)	18 (4.5)	2 (0.5)	4 (1.0)	258 (64.0)	138 (34.2)	6 (1.5)	0 (0.0)	1 (0.2)	**<0.001**
51-80 days	18 (24.0)	48 (64.0)	9 (12.0)	0 (0.0)	0 (0.0)	32 (42.7)	43 (57.3)	0 (0.0)	0 (0.0)	0 (0.0)	**<0.001**
81 days and over	74 (38.5)	97 (50.5)	16 (8.3)	5 (2.6)	0 (0.0)	121 (63.0)	66 (34.4)	5 (2.6)	0 (0.0)	0 (0.0)	**<0.001**
Place of residence											
urban area	156 (60.2)	94 (36.3)	8 (3.1)	1 (0.4)	0 (0.0)	183 (70.7)	75 (29.0)	1 (0.4)	0 (0.0)	0 (0.0)	**<0.001**
rural area	255 (47.6)	231 (43.1)	40 (7.5)	6 (1.1)	4 (0.7)	316 (59.0)	209 (39.0)	10 (1.9)	0 (0.0)	1 (0.2)	**<0.001**

Statistically significant values are bolded.

### 3.2 Changes in the prevalence of cognitive disorders and dementia among patients covered by care in day medical care homes

Among patients covered by care in Day Medical Care Homes, the percentage of patients with dementia or cognitive disorders measured with the MMSE score decreased from 54.3% on admission to 40.5% on discharge (p<0.001) (**Table 4**). Improvement in cognitive functions measured with the MMSE (p<0.05) was observed in all sociodemographic groups as well as regardless of the duration of care in the Day Medical Care Home (**Table 4**). There was no statistically significant correlation between duration of stay and absolute changes in MMSE score (rho=-0.04; p=0.3).

**Table 4:** Changes in the MMSE score of patients covered by care in Day Medical Care Homes (n=949).

Variable	MMSE score on admission					MMSE score on discharge					
	profound dementia (0-10)	medium-grade dementia (11-18)	mild dementia (19-23)	cognitive disorders without dementia (24-26)	no dementia or cognitive disorders (27-30)	profound dementia (0-10)	medium-grade dementia (11-18)	mild dementia (19-23)	cognitive disorders without dementia (24-26)	no dementia or cognitive disorders (27-30)	p
**Overall**	5 (0.5)	46 (4.8)	159 (16.8)	305 (32.1)	434 (45.7)	3 (0.3)	31 (3.3)	86 (9.1)	264 (27.8)	565 (59.5)	**<0.001**
Sex											
women	4 (0.6)	30 (4.1)	104 (14.3)	232 (31.9)	357 (49.1)	3 (0.4)	19 (2.6)	56 (7.7)	193 (26.5)	456 (62.7)	**<0.001**
Men	1 (0.5)	16 (7.2)	55 (24.8)	73 (32.9)	77 (34.7)	0 (0.0)	12 (5.2)	30 (13.5)	71 (32.0)	109 (49.1)	**<0.001**
Age											
24-59	0 (0.0)	1 (3.1)	5 (15.6)	10 (31.3)	16 (50.0)	0 (0.0)	1 (3.1)	1 (3.1)	7 (21.9)	23 (71.9)	**<0.001**
60-69	0 (0.0)	2 (0.9)	20 (9.0)	80 (36.2)	119 (53.8)	1 (0.5)	0 (0.0)	11 (5.0)	62 (28.1)	147 (66.5)	**<0.001**
70-79	2 (0.5)	10 (2.3)	58 (13.6)	134 (31.3)	224 (52.3)	1 (0.2)	7 (1.6)	23 (5.4)	102 (23.8)	295 (68.9)	**<0.001**
80-89	3 (1.2)	28 (11.3)	68 (27.4)	78 (31.5)	71 (28.6)	1 (0.4)	19 (7.7)	44 (17.7)	88 (35.5)	96 (38.7)	**<0.001**
90+	0 (0.0)	5 (25.0)	8 (40.0)	3 (15.0)	4 (20.0)	0 (0.0)	4 (20.0)	7 (35.0)	5 (25.0)	4 (20.0)	**0.002**
Duration of care											
up to 40 days	3 (2.2)	6 (4.4)	29 (21.5)	35 (25.9)	62 (45.9)	1 (0.7)	7 (5.2)	9 (6.7)	36 (26.7)	82 (60.7)	**<0.001**
41-50 days	2 (0.4)	17 (3.5)	83 (17.0)	147 (30.1)	240 (49.1)	1 (0.2)	9 (1.8)	47 (9.6)	135 (27.6)	297 (60.7)	**<0.001**
51-80 days	0 (0.0)	3 (3.2)	7 (7.4)	50 (53.2)	34 (36.2)	1 (1.1)	2 (2.1)	3 (3.2)	19 (20.2)	69 (73.4)	**<0.001**
81 days and over	0 (0.0)	20 (8.7)	40 (17.3)	73 (31.6)	98 (42.4)	0 (0.0)	13 (5.6)	27 (11.7)	74 (32.0)	117 (50.6)	**<0.001**
Place of residence											
urban area	0 (0.0)	20 (5.6)	74 (20.8)	96 (27.0)	165 (46.5)	0 (0.0)	14 (3.9)	42 (11.8)	88 (24.8)	211 (59.4)	**<0.001**
rural area	5 (0.8)	26 (4.4)	85 (14.3)	209 (35.2)	269 (45.3)	3 (0.5)	17 (2.9)	44 (7.4)	176 (29.6)	354 (59.6)	**<0.001**

Statistically significant values are bolded.

## 4 Discussion

Day Medical Care Home is a model of day care for patients with disabilities in Poland, co-funded by the EU and operating in Poland between 2015 and 2023 (15, 16). This is the first study to assess the impact of Day Medical Care Homes on the mental health status and cognitive functions of patients admitted to Day Medical Care Homes. On discharge, when compared to patients’ status on admission, significant improvement in the prevalence of depressive symptoms and cognitive functions was observed in all socio-demographic groups.

Population aging, limited healthcare resources, and growing expenditures on healthcare prompted public authorities to develop new forms of care for older patients, including patients with disabilities. Day care services are widely implemented in psychiatry as an effective model of care for patients with selected mental health issues (24, 25). Day care models were also implemented as a form of care for older adults (26).

Findings from this study showed, that patients covered by day care offered in Day Medical Care Homes achieved an improvement in mental health status, measured with the GDS-15 scale and HAM-D scale. Depression is a complex mental health illness, with various causes and clinical course (27, 28). Patients admitted to Day Medical Care Homes were covered by the care provided by occupational therapists, and psychologists or psychotherapists which may have the highest impact on the improvement in mental health status. Moreover, patients spend at least 4 hours in a group, so meeting with other people and community networks may have an impact on a decrease in the percentage of patients with depression on discharge (29). Findings from this study are in line with data on improvement in the mental health status of patients covered by psychiatric day care services (24, 25). When measured with HAM-D score, longer duration of stay was significantly correlated with the greatest reduction in the depression score on discharge. Further analysis may identify groups of patients that will benefit from longer stays in Day Medical Care Homes.

In this study, patients discharged from Day Medical Care Homes also noted improvement in cognitive function measured with the MMSE scale. Motor improvement (rehabilitation) and stimulation of cognitive processes were services provided in Day Medical Care Homes. Rehabilitation, including physiotherapy and kinesitherapy, may have the greatest impact on the improvement of cognitive functions of patients (30). However, patients admitted to the Day Medical Care Homes were educated on compliance and adherence to treatment guidelines and rules of self-care in chronic diseases, which may have also an impact on cognitive functions (31, 32). Diet, the need to follow a schedule and daily plan, participation in occupational therapy, and contact with other people (socialization) could also improve cognitive functions (33).

In this study, significant improvement in mental health status and cognitive functions was observed regardless of sex, age, place of residence, and duration of care in Day Medical Care Home. This finding suggests that the scope of services offered in Day Medical Care Homes was sufficient to address the health and social needs of different socio-demographic groups, and this model of day care can be multiplicated and new Day Medical Care Homes may be developed in different locations.

This is the first study on comparisons of mental health status and cognitive function on admission and discharge among patients admitted to Day Medical Care Homes in Poland, so different comparisons with other studies are not possible.

This study has practical implications for public policies on senior care. Findings from this study confirmed that Day Medical Care Home was an effective form of day care that led to improvement in mental health status as well as cognitive functions of older patients with disabilities covered by this form of day care. Moreover, lessons learned from the implementation of Day Medical Care Homes in Poland and data on their impact on patients’ status, may be used by other countries to implement a similar organizational model of day care for older adults. Findings from this study may also pose a basis for benchmarking with another form of care on older patients with disabilities. Findings from this study also underline the need to perform longitudinal follow-up studies to verity the impact of this senior care model on the functional and mental health status of patients in longer time-periods after discharge.

This study was limited to 3 scales (2 on depression and 1 on cognitive functions) which is a major limitation of this study. However, the range of data collected was defined by funding institutions (Ministry of Health under the EU program). Data on comorbidities and medicines were not available for the analysis, as all data were collected using paper-based forms. Electronic records on comorbidities and medication use were not available, which is a limitation of this study. Third, this study is limited to seven Day Medical Care Homes managed by one company (the Medical and Diagnostic Centre). Another potential limitation in the generalizability of the findings due to the specific sample. The study’s observational nature may limit causal inferences. The MMSE questionnaire was originally validated for the assessment of Alzheimer’s-type dementias, and its use in this study was based on common practice in other studies. Nevertheless, this is the first study on the mental health status and cognitive functions of patients covered by this new model of day care and further studies from other Day Medical Care Homes may provide more comprehensive data.

## 5 Conclusions

Day Medical Care Home is an effective model of day care that improves the mental health status and cognitive functions of older patients with disabilities. Improvement in mental health status and cognitive functions were achieved in all demographic groups, regardless of sex, age, duration of care, and place of residence, which suggests the model of Day Medical Care Homes may have high potential for implementation in different local communities.

## Data availability statement

The raw data supporting the conclusions of this article will be made available by the authors, without undue reservation.

## Ethics statement

The studies involving humans were carried out following guidelines expressed in the Declaration of Helsinki and the protocol was approved by the Ethics Committee at the Medical University of Warsaw, Poland (approval number: AKBE/298/2023; 13 Nov 2023). The studies were conducted in accordance with the local legislation and institutional requirements. The participants provided their written informed consent to participate in this study.

## Author contributions

MJ: Conceptualization, Formal analysis, Methodology, Writing – original draft, Writing – review & editing. LW: Conceptualization, Data curation, Formal analysis, Investigation, Methodology, Project administration, Visualization, Writing – original draft, Writing – review & editing. AA: Data curation, Investigation, Writing – original draft, Writing – review & editing. MG: Conceptualization, Investigation, Methodology, Resources, Supervision, Writing – original draft, Writing – review & editing.
